# Blockade of cholinergic transmission elicits somatic signs in nicotine-naïve adolescent rats

**DOI:** 10.3389/fphar.2015.00239

**Published:** 2015-10-20

**Authors:** Clare E. Schmidt, Katherine E. Manbeck, David Shelley, Andrew C. Harris

**Affiliations:** ^1^Minneapolis Medical Research Foundation, Minneapolis, MN, USA; ^2^Department of Neuroscience, University of Minnesota, Minneapolis, MN, USA; ^3^Department of Psychology, University of Minnesota, Minneapolis, MN, USA; ^4^Department of Medicine, University of Minnesota, Minneapolis, MN, USA

**Keywords:** mecamylamine, nicotine, adolescents, rat, somatic signs

## Abstract

High doses of the nicotinic acetylcholine receptor (nAChR) antagonist mecamylamine can elicit somatic signs resembling those associated with nicotine withdrawal in nicotine-naïve adult rats. Understanding this phenomenon, and its possible modulation by acute nicotine and age, could inform the use of mecamylamine as both an experimental tool and potential pharmacotherapy for tobacco dependence and other disorders. This study evaluated the ability of high-dose mecamylamine to elicit somatic signs in adolescent rats, and the potential for acute nicotine pretreatment to potentiate this effect as previously reported in adults. Single or repeated injections of mecamylamine (1.5 or 3.0 mg/kg, s.c.) elicited somatic signs in nicotine-naïve adolescents, but this effect was not influenced by 2 h pretreatment with acute nicotine (0.5 mg/kg, s.c.). In an initial evaluation of the effects of age in this model, mecamylamine (2.25 mg/kg, s.c.) elicited somatic signs in nicotine-naïve adolescents and adults. This effect was modestly enhanced following acute nicotine injections in adults but not in adolescents, even when a higher nicotine dose (1.0 rather than 0.5 mg/kg, s.c.) was used in adolescents to account for age differences in nicotine pharmacokinetics. These studies are the first to show that mecamylamine elicits somatic signs in nicotine-naïve adolescent rats, an effect that should be considered when designing and interpreting studies examining effects of high doses of mecamylamine in adolescents. Our findings also provide preliminary evidence that these signs may be differentially modulated by acute nicotine pretreatment in adolescents versus adults.

## Introduction

The non-selective nicotinic acetylcholine receptor (nAChR) antagonist mecamylamine is commonly used in preclinical models. For example, in a well-established model of antagonist-precipitated nicotine withdrawal, low doses of mecamylamine elicit increases in somatic (physical) signs (e.g., abdominal constrictions, facial fasciculations) in rats receiving a chronic nicotine infusion without affecting somatic signs in nicotine-naïve rats (e.g., Malin et al., 1994; Watkins et al., 2000; Malin and Goyarzu, 2009). At high doses, mecamylamine can itself elicit somatic signs in nicotine-naïve rats that resemble those associated with nicotine withdrawal (Malin et al., 1994; Harrison et al., 2001; Wing and Shoaib, 2007; Guillem et al., 2008; Harris et al., 2013), as well as other behavioral effects including suppression of operant responding (Levin et al., 2000; Vann et al., 2006), cognitive effects (Levin et al., 1987, 2000; Sanders et al., 2010), and aversion (Watkins et al., 2000; Guillem et al., 2008). These effects of mecamylamine alone presumably reflect antagonism of endogenous nAChRs, which are prominently expressed in numerous brain areas that mediate behavior including the ventral tegmental area, hippocampus, medial habenula, and interpeduncular nucleus (Wada et al., 1989; Gotti et al., 2006, 2009; Fowler et al., 2008).

Understanding the effects of high doses of mecamylamine alone on somatic signs and other behaviors is important for several reasons. First, these effects can complicate data interpretation in preclinical models, such as when mecamylamine is being used to modulate the effects of nicotine. Second, these effects may be relevant to mecamylamine’s adverse side effects in humans (e.g., constipation, dizziness) that limit mecamylamine’s use as a potential treatment for tobacco dependence and other disorders (e.g., Tourette’s syndrome) in humans (Shytle et al., 2002; Bacher et al., 2009; Nickell et al., 2013). In addition to informing the use of mecamylamine as both an experimental tool and putative pharmacotherapy, studying the behavioral effects of mecamylamine alone could also provide important basic scientific knowledge on the function of the cholinergic system.

We recently reported that acute nicotine pretreatment enhanced the ability of high doses of mecamylamine to elicit somatic signs in adult rats (Harris et al., 2013). Given that increased sensitivity to the effects of an antagonist in subjects acutely exposed to drugs is classically interpreted as a withdrawal effect (e.g., Adams and Holtzman, 1990; Easterling and Holtzman, 1997; Schulteis et al., 1997; Cunningham et al., 2014), this phenomenon may reflect the early development of the nicotine withdrawal syndrome (Harris et al., 2013). Alternatively, it could reflect a nicotine-mecamylamine interaction unrelated to withdrawal. Regardless of its interpretation, this effect is of interest because mecamylamine exposure occurs in the presence of acute nicotine exposure in animal models (Cohen et al., 2003; Liu et al., 2007; Struthers et al., 2009) and in human smokers (McClernon and Rose, 2005; McKee et al., 2009).

It is well established that adolescent and adult rodents can differ in their response to pharmacological manipulation of nAChRs (e.g., Shram et al., 2006, 2008a; Torres et al., 2008). For example, adolescents exhibit attenuated somatic signs compared to adults during mecamylamine-precipitated withdrawal from a chronic nicotine infusion (O’Dell et al., 2004, 2006; Shram et al., 2008b). To our knowledge, no studies have evaluated effects of high-dose mecamylamine on somatic signs in drug-naïve adolescent rats, or modulation of these effects by acute nicotine pretreatment.

The primary goal of this study was to evaluate effects of acute mecamylamine, nicotine, and their combination on somatic signs in adolescent rats using the same protocol previously studied in adults (Harris et al., 2013). A secondary goal was to examine age differences in these effects. Therefore, following establishment of nicotine dosing conditions that accounted for age differences in nicotine pharmacokinetics (see Vieira-Brock et al., 2013; Craig et al., 2014) and produced similar nicotine serum levels across ages (Experiment 2), we evaluated effects of mecamylamine alone and mecamylamine combined with acute nicotine on somatic signs in both adolescents and adults (Experiment 3).

## Materials and Methods

### Animals

Experimentally naïve male Wistar rats (Charles River Laboratories, Wilmington, MA, USA) were housed individually in a temperature- and humidity-controlled colony room under a reversed 12-h light/dark cycle with free access to food and water. Upon arrival in the colony, adult rats (PND 62–67) weighed 275–300 g and adolescent rats (PND 21–23) weighed < 50 g. Rats were allowed a period of 2 weeks to acclimate to the experimental housing after arrival and were gently handled for approximately 5 min on each of 2 days before all experiments. All testing was conducted during the dark (active) phase. With the exception of the challenge test in Experiment 1, all procedures in adolescent rats were conducted from PND 35–42 (i.e., during mid- adolescence, Spear, 2000), the same age range used in (O’Dell et al., 2004, 2006; Shram et al., 2008b). The age range for adults in the current studies (i.e., PND 76–83) was also similar to that used in these studies. All procedures were approved by The Institutional Animal Care and Use Committee (IACUC) of the Minneapolis Medical Research Foundation (protocol # 08–08) in accordance with the 2011 NIH Guide for the Care and Use of Laboratory Animals and the 2003 Guidelines for the Care and Use of Mammals in Neuroscience and Behavioral Research. All efforts were made to minimize animal suffering.

### Drugs

Nicotine bitartrate or mecamylamine hydrochloride (Sigma Chemical Co., St. Louis, MO, USA) was dissolved in sterile saline. The pH of the nicotine solution was adjusted to 7.4 using NaOH. Nicotine and mecamylamine doses are expressed as the base and salt, respectively. All injections were administered s.c. in a volume of 1.0 ml/kg.

### Experiment 1: Effects of Mecamylamine, Nicotine, and Their Combination on Somatic Signs in Adolescents

#### Protocol

The procedure was identical to that previously used to study the effects of acute mecamylamine, nicotine, and their combination on somatic signs in adult rats (Harris et al., 2013). On the first test day, adolescent rats (PND 35–37) were injected with saline (Sal) or 0.5 mg/kg nicotine (Nic 0.5). 1 h 50 min after the first injection, animals were injected with 0, 1.5, or 3.0 mg/kg mecamylamine (i.e., Sal, Mec 1.5, or Mec 3.0) and, 10 min later, tested for somatic signs as described below. This procedure was repeated across 5 consecutive days, with rats receiving the same treatment each day. To examine the persistence of any sensitization-like effects observed during the 5-day protocol, rats were re-tested under the same conditions following a 7-day drug-free period (challenge test). The following 6 groups were used: Nic 0.5 + Mec 3.0 (*n* = 4), Nic 0.5 + Mec 1.5 (*n* = 6), Nic 0.5 + Sal (*n* = 6), Sal + Mec 3.0 (*n* = 4), Sal + Mec 1.5 (*n* = 5), Sal + Sal (*n* = 6).

#### Assessment of Somatic Signs

During each test session, rats were placed in a clear plastic container located in a quiet, lit room and videotaped for 10 min. Test sessions were later scored for somatic signs by a trained, blinded observer using a validated checklist as described previously (Harris et al., 2011, 2013; Manbeck et al., 2014). Individual categories of somatic signs included abdominal constrictions (writhes and gasps), shakes and tremors, blinks, and other miscellaneous signs including facial fasciculation, yawns, and ptosis. If present continuously, facial fasciculations were scored once every 15 s and ptosis once per minute.

### Experiment 2: Serum and Brain Nicotine Levels in Adolescents and Adults Following Acute Nicotine

#### Protocol

Adolescent (PND 35–37) or adult (PND 76–81) rats were injected with 0.5 mg/kg nicotine (*n* = 8 per age group) or 1.0 mg/kg nicotine (adolescents only, *n* = 8). 1 h 50 min later, rats were anesthetized with i.m. droperidol (2.0 mg/kg)/fentanyl (0.04 mg/kg). 10 min later, rats were decapitated and trunk blood and brain were collected. Timing of sample collection (relative to nicotine injection) coincided with the timing of somatic sign testing in Experiments 1 and 3.

#### Nicotine Assay

Serum and brain nicotine levels were measured using gas chromatography with nitrogen-phosphorous detection (Jacob et al., 1981). Brain nicotine levels were corrected for brain blood content (Hieda et al., 1999).

### Experiment 3: Effects of Mecamylamine Alone and Mecamylamine Combined with Nicotine on Somatic Signs in Adolescents and Adults

**FIGURE 1:**
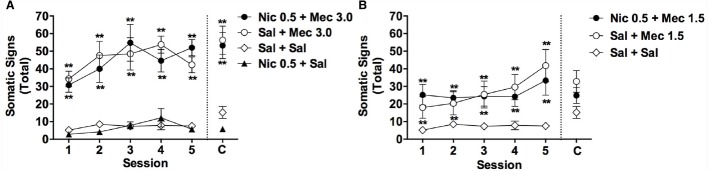
**Effects of acute mecamylamine, nicotine, and their combination on somatic signs in adolescents.** Mean (±SEM) total number of somatic signs during each test session in Experiment 1. For clarity, data from the Nic + Mec 1.5 and Sal + Mec 1.5 groups are graphed separately in **(B)**. Data from the Sal + Sal group are included in both **(A)** and **(B)**. C = Challenge test conducted 1 week after session 5. **Significantly different from Sal+ Sal group, *p* < 0.01.

Adolescent (PND 35–37) and adult (PND 76–81) rats were injected with saline, 0.5 mg/kg nicotine (adults only), or 1.0 mg/kg nicotine (adolescents only). 1 h 50 min later, rats were administered mecamylamine (0 or 2.25 mg/kg, s.c.) and, 10 min later, tested for somatic signs as described above. This procedure was repeated 48 h later (2 test sessions total). These nicotine doses were used because they produced similar serum nicotine levels in adolescents and adults in Experiment 2. We previously found that acute nicotine pretreatment potentiated the effects of 3.0 mg/kg mecamylamine on somatic signs in adults (Harris et al., 2013), We used a lower (2.25 mg/kg) dose of mecamylamine in this study an attempt to avoid the robust effects of 3.0 mg/kg mecamylamine in drug-naïve adolescents in Experiment 1 (see Sal + Mec 3.0 group in Figure 1A), which could create a ceiling effect that precluded potentiation of these effects following nicotine pretreatment. Furthermore, pilot studies indicated that acute nicotine pretreatment potentiated the effects of 2.25 mg/kg mecamylamine on somatic signs in adults (data not shown), supporting the use of this mecamylamine dose for studying nicotine-mecamylamine interactions. We used a 2-day procedure to expedite the protocol and to avoid the trend for the effects of mecamylamine alone to increase in adolescents when a 5-day procedure was used (see Figures 1A,B), which could also produce a ceiling effect. Adolescents were tested in the following groups: Nic 1.0 + Mec 2.25 (*n* = 6), Sal + Mec 2.25 (*n* = 6), Nic 1.0 + Sal (*n* = 5), Sal + Sal (*n* = 6). Experimental groups for adults were Nic 0.5 + Mec 2.25 (*n* = 9), Sal + Mec 2.25 (*n* = 9), Sal + Sal (*n* = 6). A Nic 0.5 + Sal adult control group was not included because we have previously shown that 2 h pretreatment with 0.5 mg/kg nicotine alone does not affect somatic signs in adults (Harris et al., 2013).

### Statistical Analyses

#### Experiment 1

Total somatic signs during test sessions 1–5 were analyzed using a two-factor ANOVA with group as a between-subject factor and session as a within-subject factor, followed by Bonferroni *post hoc* tests for subsequent between-group comparisons. To evaluate any changes (e.g., sensitization) in drug effects across sessions 1–5, data within each group were also analyzed using a one-factor, repeated measures ANOVA. Data from the challenge test were analyzed using a single-factor ANOVA followed by Bonferroni *post hoc* tests for between-group comparisons. Paired-samples *t*-tests were also used to compare data from the challenge test to data from session 1 for each group. In this and other experiments, *p*-values ≤ 0.05 were considered statistically significant.

#### Experiment 2

Serum nicotine concentrations and brain: serum nicotine concentration ratios were analyzed using separate one-way ANOVAs with group as a factor, followed by Bonferroni *post hoc* tests comparing the adult group administered 0.5 mg/kg nicotine to the adolescent groups administered 0.5 mg/kg or 1.0 mg/kg nicotine. Data for brain nicotine concentrations were not normally distributed and were therefore analyzed using a one-way Kruskal-Wallace test followed by Dunn’s *post hoc* test.

#### Experiment 3

Total somatic signs during sessions 1 and 2 in adults and adolescents were analyzed using a three-factor ANOVA with age and group as between-subject factors, and session as a within-session factor. Data for each age were subsequently analyzed using two-factor (group × session) ANOVAs followed by Bonferroni *post hoc* tests for comparisons between groups.

To evaluate the effects of nicotine alone on somatic signs in adolescents, data for the adolescent Nic 1.0 + Sal group were compared to data from the adolescent Sal + Sal group using a two-factor (group × session) ANOVA followed by a Bonferroni *post hoc* test for comparisons between groups. This analysis could not be included in the between-age comparison described above because a group of adults receiving nicotine alone was not tested.

## Results

### Experiment 1: Effects of Acute Mecamylamine, Nicotine, and Their Combination on Somatic Signs in Adolescents

#### Sessions 1–5

Single or repeated injections of mecamylamine alone (1.5 or 3.0 mg/kg, s.c.) elicited somatic signs in adolescent rats, but this effect was not influenced by 2 h pretreatment with nicotine (0.5 mg/kg, s.c.; Figures 1A,B). There were significant main effects of group [*F*(5,25) = 19.3, *p* < 0.0001] and session [*F*(4,104) = 6.7, *p* < 0.0001] on somatic signs during sessions 1–5, but no significant group × session interaction. Somatic signs were elevated in the Nic 0.5 + Mec 3.0, Sal + Mec 3.0, Nic 0.5 + Mec 1.5, and Sal + Mec 1.5 groups compared to the Sal + Sal group across sessions 1–5 (Bonferroni *t* = 3.8–6.9, *p*s < 0.01). No other significant differences between groups were observed.

A one-factor, repeated measures ANOVA within each group indicated a trend toward an effect of session for the Sal + Mec 1.5 group [*F*(4,16) = 2.9, *p* = 0.057] and the Sal + Mec 3.0 group [*F*(4,16) = 2.5, *p* = 0.098], reflecting a tendency for the effects of mecamylamine alone to increase across repeated injections (see Figures 1A,B). There was no effect of session in any other group.

#### Challenge Test

Due to malfunction of the video recording equipment, data were not available for three rats (one each from the Sal + Sal, Nic 0.5 + Sal, and Nic + Mec 1.5 groups). Analysis of data from the remaining animals indicated a significant effect of group during the challenge test [*F*(5,52) = 14.0, *p* < 0.0001]. The Nic 0.5 + Mec 3.0 and Sal + Mec 3.0 groups exhibited a greater number of signs than the Sal + Sal group (*t* = 5.0 or 5.4, *p*s < 0.01; Figure 1A), but did not differ from each other. No other group differed significantly from the Sal + Sal group (Figures 1A,B). Somatic signs during the challenge test did not differ from those during session 1 for any group (all *p*-values > 0.05).

### Experiment 2: Serum and Brain Nicotine Levels in Adolescents and Adults Following Acute Nicotine

**FIGURE 2:**
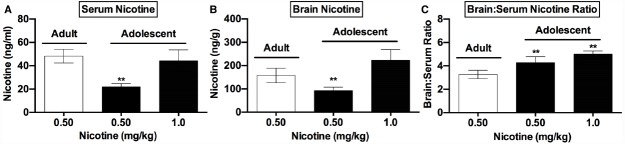
**Nicotine levels in adolescents and adults following acute nicotine.** Mean (±SD) serum **(A)** and brain **(B)** nicotine concentrations and brain:serum nicotine concentration ratios **(C)** in adult and adolescent rats in Experiment 2. **Significantly different from adults receiving 0.5 mg/kg nicotine, *p* < 0.01.

Analysis of data from adults administered 0.5 mg/kg nicotine and adolescents administered 0.5 or 1.0 mg/kg nicotine indicated significant effects of group on serum [*F*(5,21) = 37.3, *p* < 0.0001] and brain (Kruskal-Wallis H = 18.5, *p* < 0.001) nicotine levels collected 2 h post-injection. Serum and brain nicotine levels were lower in adolescents receiving 0.5 mg/kg nicotine compared to those in adults receiving the same dose (*p* < 0.05 or 0.01; Figures 2A,B). In contrast, serum nicotine levels in adolescents administered 1.0 mg/kg nicotine did not differ from those in adults administered 0.5 mg/kg nicotine (Figure 2A). Brain nicotine levels in adolescents administered 1.0 mg/kg nicotine were slightly higher than those in adults administered 0.5 mg/kg nicotine (see Figure 2B), but this difference was not significant (*p* = 0.10). There was also a significant difference in brain:serum nicotine concentration ratios between groups [*F*(5,21) = 39.9, *p* < 0.0001], with a higher ratio in both adolescent groups compared to the adult group (Figure 2C, *t* = 5.2, or 8.9, *p* < 0.01).

### Experiment 3: Effects of Acute Mecamylamine, Nicotine, and Their Combination on Somatic Signs in Adolescents and Adults

#### Effects of Mecamylamine Alone and Mecamylamine Combined with Nicotine on Somatic Signs in Adolescents and Adults

An initial three-factor ANOVA indicated significant effects of age [*F*(1,36) = 7.7, *p* < 0.01] and group [*F*(2,36) = 41.4, *p* < 0.0001] on somatic signs, but no significant effects of session or interactions.

**FIGURE 3:**
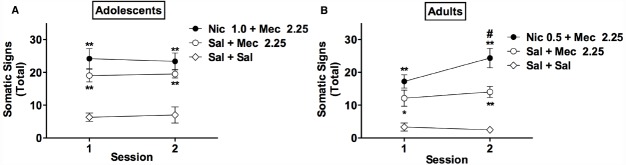
**Effects of mecamylamine alone and mecamylamine combined with nicotine on somatic signs in adolescents and adults.** Mean (±SEM) total number of somatic signs during each test session in adolescents **(A)** and adults **(B)** in Experiment 3. ^*^^,^^**^Significantly different from Sal+ Sal group for that age, *p* < 0.05, 0.01. # Adult Nic 0.5 + Mec 2.25 group significantly different from adult Sal + Mec 2.25 group at that session, *p* < 0.01.

A subsequent two-factor (group × session) ANOVA on data for adolescents indicated a significant main effect of group [*F*(3,19) = 40.7, *p* < 0.0001], but no effect of session or group × session interaction (Figure 3A). Somatic signs were higher in the Nic 1.0 + Mec 2.25 and Sal + Mec 2.25 groups compared to the adolescent Sal + Sal group (*t* = 5.8–9.4, *p* < 0.01), but these groups did not differ from one another (Figure 3A).

In adults, there were significant main effects of group [*F*(2,21) = 19.5, *p* < 0.0001] and session [*F*(1,21) = 4.9, *p* < 0.05] on somatic signs, and a significant group × session interaction [*F*(2,21) = 3.6, *p* < 0.05; Figure 3B]. Somatic signs were elevated in the Nic 0.5 + Mec 2.25 and Sal + Mec 2.25 groups compared to the adult Sal + Sal group during sessions 1 and 2 (*t* = 2.7–6.7, *p* < 0.05, or 0.01; Figure 3B). Somatic signs were also higher in the Nic 0.5 + Mec 2.25 group compared to the Sal + Mec 2.25 group during session 2 (*t* = 3.4, *p* < 0.01).

In summary, mecamylamine (2.25 mg/kg) elicited robust somatic signs in nicotine-naïve adolescents and adults. This effect was modestly enhanced by acute nicotine pretreatment in adults but not in adolescents, even when adolescents were administered a higher nicotine dose (1.0 rather than 0.5 mg/kg, s.c.) to account for age differences in nicotine pharmacokinetics.

#### Effects of Nicotine Alone in Adolescents

Comparison of data for the adolescent Nic 1.0 + Sal group (mean ± SEM signs during sessions 1 and 2 = 1.8 ± 0.6 and 2.8 ± 1.0, respectively) and the adolescent Sal + Sal group (data shown in Figure 1A) indicated a significant effect of group [*F*(1,9) = 9.0, *p* < 0.05], but no effect of session or group × session interaction. The two groups did not differ significantly from one another at either session (*p* > 0.13).

## Discussion

This study evaluated the ability of high-dose mecamylamine to elicit somatic signs in nicotine-naïve adolescent rats, and the potential for acute nicotine pretreatment to potentiate this effect as previously reported in adults. In Experiment 1, mecamylamine (1.5 or 3.0 mg/kg) elicited somatic signs in adolescents, but this effect was not potentiated by 2 h pretreatment with acute nicotine (0.5 mg/kg). In Experiment 2, 2 h pretreatment with 1.0 mg/kg nicotine produced serum nicotine levels in adolescents that were similar to those observed in adults administered 0.5 mg/kg nicotine, thereby identifying appropriate nicotine doses for an initial age comparison. In Experiment 3, mecamylamine (2.25 mg/kg) elicited somatic signs in adolescents and adults. This effect was modestly enhanced in adults administered 0.5 mg/kg nicotine, but not in adolescent rats administered 1.0 mg/kg nicotine.

These studies are the first to examine the effects of acute mecamylamine, nicotine, and their combination on somatic signs in adolescent rats. While such effects have previously been studied in adults (Harris et al., 2013), our findings could not be predicted based on these data given the important age differences in the effects of pharmacological manipulation of nAChRs (e.g., Shram et al., 2006, 2008a; Torres et al., 2008). Our findings emphasize the need to consider the presence of somatic signs when designing and interpreting studies involving administration of high mecamylamine doses to adolescent rodents. This phenomenon would most obviously impact studies in which somatic signs are used to measure mecamylamine-precipitated withdrawal in rats receiving a chronic nicotine infusion, but could also impact behavior in other models (see Malin et al., 1994). Furthermore, our findings suggest that the effects of mecamylamine on somatic signs in adolescents should be equally considered regardless of whether animals are also exposed to acute nicotine.

Mecamylamine is being evaluated as a treatment for tobacco dependence and other disorders in clinical populations that include adolescents and young adults (Shytle et al., 2002; Bacher et al., 2009; Nickell et al., 2013). To the extent that effects of mecamylamine alone on somatic signs are relevant to mecamylamine’s adverse side effects in humans (Harris et al., 2013), our findings raise the possibility that these effects may be potentiated by concurrent nicotine exposure in adults but not adolescents. Further use of these models could lead to new approaches for reducing mecamylamine’s side effects and facilitating its use as a putative pharmacotherapy.

In addition to acting as a non-selective antagonist at nAChRs, mecamylamine can have other neurobiological effects including blockade of glutamatergic *N*-methyl-D-aspartate (NMDA) receptors (Omori et al., 1988; Papke et al., 2001) or increases in brain serotonin levels (Kenny et al., 2000; Ma et al., 2006). It is unlikely that these effects account for mecamylamine’s elevation of somatic signs, however, as NMDA receptor antagonists and serotonin agonists do not elicit somatic signs in drug-naïve rats (Fundytus and Coderre, 1994; Harrison et al., 2001) and can actually *suppress* somatic signs during withdrawal from chronic exposure to nicotine or other drugs (Higgins et al., 1992; Fundytus and Coderre, 1994; Ohmura et al., 2011). The ability of mecamylamine to elicit somatic signs in nicotine-naïve rodents therefore most likely reflects antagonism of endogenous nAChRs, at least in part.

We reported that the effects of 3.0 mg/kg mecamylamine on somatic signs in adults were enhanced following a single nicotine injection (0.5 mg/kg, s.c.) (Harris et al., 2013). Although a significant enhancement of the effects of 2.25 mg/kg mecamylamine on some individual categories of signs (e.g., abdominal constrictions) occurred in adults following a single nicotine injection in Experiment 3 (data not shown), reliable enhancement of mecamylamine’s effects on *total* somatic signs were only observed after a second nicotine injection (see Figure 3B). While the use of different mecamylamine doses across studies represents the most obvious explanation for this discrepancy, it could also reflect variability within the model due to cohort effects, reliance on an observer-rated measure, or other factors. Regardless, both data sets show that mecamylamine’s effects on somatic signs can be modestly potentiated following a limited number of acute nicotine injections in adult rats.

We have proposed that an enhancement of mecamylamine’s effects on somatic signs following acute nicotine exposure may be relevant to nicotine withdrawal (Harris et al., 2013), similar to how other authors have interpreted increased antagonist sensitivity following acute exposure to other drugs (e.g., morphine; Adams and Holtzman, 1990; Easterling and Holtzman, 1997; Schulteis et al., 1997, 1999). In support of this interpretation, the diminished sensitivity of adolescents to the effects of nicotine in the current models parallels the diminished withdrawal sensitivity of adolescents in traditional models involving chronic nicotine infusion (O’Dell et al., 2004, 2006; Shram et al., 2008b). Furthermore, the dosing regimen used for the Nic + Mec 3.0 group in Experiment 1 elicits elevations in intracranial self-stimulation thresholds (anhedonia-like behavior) in adult rodents (Harris et al., 2013), which is a well established measure of withdrawal from chronic nicotine exposure (Epping-Jordan et al., 1998; Watkins et al., 2000; Roiko et al., 2009). Nonetheless, the relevance of the current effect to nicotine withdrawal should be confirmed using a range of validation criteria (e.g., blockade by pharmacotherapies that relieve nicotine withdrawal in smokers such as bupropion; Malin et al., 1992, 2006; Malin and Goyarzu, 2009).

Alternatively, the ability of nicotine pretreatment to enhance mecamylamine’s effects on somatic signs in adults may be unrelated to nicotine withdrawal. For example, to the extent that mecamylamine’s effects reflect general malaise or aversion (Malin et al., 1994), this phenomenon might be better described as a nicotine-induced potentiation of mecamylamine’s aversive effects. Viewed from this perspective, our demonstration of reduced sensitivity of adolescents would complement studies indicating that adolescents are relatively insensitive to the acute aversive effects of nicotine itself (Wilmouth and Spear, 2004; Shram et al., 2006; Torres et al., 2008). Further research is clearly needed to better understand the nature of the interaction between acute nicotine and mecamylamine on somatic signs.

The lower serum and brain nicotine levels in adolescents versus adults following injection of 0.5 mg/kg nicotine in Experiment 2 is consistent with recent findings (Vieira-Brock et al., 2013; Craig et al., 2014). We also found higher brain:serum nicotine concentration ratios in adolescents compared to adults, suggesting greater penetration of nicotine into brain. This phenomenon, which was also reported in (Craig et al., 2014), suggests a potential mechanism for the enhanced rewarding effects of nicotine reported in adolescent rodents in some studies (Shram et al., 2006; O’Dell, 2009). More generally, the current pharmacokinetic data emphasize the need to consider between-age differences in nicotine pharmacokinetics when comparing the effects of nicotine in adolescent and adult animals.

In conclusion, this study demonstrates that mecamylamine can elicit somatic signs in adolescent rats in either the presence or absence of acute nicotine. Further development of these models, including evaluation of age differences across a range of nicotine and mecamylamine doses and extension of these models to other behavioral effects of mecamylamine alone (e.g., suppression of operant responding), is warranted.

### Conflict of Interest Statement

The authors declare that the research was conducted in the absence of any commercial or financial relationships that could be construed as a potential conflict of interest.
